# Consensus statement on “Oral frailty” from the Japan Geriatrics Society, the Japanese Society of Gerodontology, and the Japanese Association on Sarcopenia and Frailty

**DOI:** 10.1111/ggi.14980

**Published:** 2024-10-07

**Authors:** Tomoki Tanaka, Hirohiko Hirano, Kazunori Ikebe, Takayuki Ueda, Masanori Iwasaki, Shunsuke Minakuchi, Hidenori Arai, Masahiro Akishita, Koichi Kozaki, Katsuya Iijima

**Affiliations:** ^1^ Joint Working Committee on Oral Frailty by the Japan Geriatrics Society Japanese Society of Gerodontology, and Japanese Association on Sarcopenia and Frailty Tokyo Japan; ^2^ Institute of Gerontology The University of Tokyo Tokyo Japan; ^3^ Tokyo Metropolitan Institute for Geriatrics and Gerontology Tokyo Japan; ^4^ Department of Removable Prosthodontics and Gerodontology, Graduate School of Dentistry Osaka University Osaka Japan; ^5^ Department of Removable Prosthodontics and Gerodontology Tokyo Dental College Tokyo Japan; ^6^ Department of Preventive Dentistry, Faculty of Dental Medicine and Graduate School of Dental Medicine Hokkaido University Hokkaido Japan; ^7^ Department of Gerodontology and Oral Rehabilitation, Graduate School of Medical and Dental Sciences Tokyo Medical and Dental University Tokyo Japan; ^8^ National Center for Geriatrics and Gerontology Aichi Japan; ^9^ Department of Geriatric Medicine Kyorin University School of Medicine Tokyo Japan; ^10^ Institute for Future Initiatives The University of Tokyo Tokyo Japan

**Keywords:** public health, frailty, oral frailty, oral function, oral health

## Abstract

The concept of oral frailty was first proposed in Japan in 2014 by the “Joint Working Committee on Oral Frailty,” consisting of three academic societies—the Japan Geriatrics Society, the Japanese Society of Gerodontology, and the Japanese Association on Sarcopenia and Frailty—to enhance public understanding of oral frailty. Oral frailty is a state between robust oral function (a “healthy mouth”) and its decline, characterized by slight declines in oral function, including tooth loss and difficulties in eating and communicating, which increase the risk of impaired oral functional capacity but can be reversed with proper intervention and treatment. Oral frailty can be assessed using the Oral Frailty 5‐item Checklist (OF‐5) without the need for a dental health professional. Oral frailty is defined as having at least two of the following components: (i) fewer teeth, (ii) difficulty chewing, (iii) difficulty swallowing, (iv) dry mouth, and (v) low articulatory oral motor skills. Approximately 40% of community‐dwelling older adults have oral frailty. Oral frailty is associated with poor dietary variety, social isolation, physical frailty, disability, and mortality. This statement introduces the concept and definition of oral frailty, a new assessment tool (OF‐5), and concept diagrams for healthcare professionals and the general public. These tools aim to promote public awareness and facilitate collaboration between medical and dental healthcare providers. **Geriatr Gerontol Int 2024; 24: 1111–1119**.

## Graphical abstract

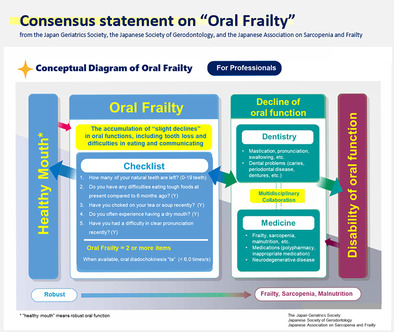

## Issues surrounding the aging society in Japan

Japan is experiencing an unprecedented phase of super‐aging, with the highest global aging rate and the onset of a centenarian era. The older adult population is projected to peak after 2040, particularly for those aged ≥75 years, with declining birth rates and a shrinking working‐age population.^1^ To achieve a sustainable and secure society of centenarians, preserving their physical and mental autonomy is crucial. Measures for preventing frailty, including oral frailty, should be implemented to prolong healthy life expectancy.^2^ There is a need to raise public awareness, as well as collaborative efforts across industry, academia, government, and various disciplines, to enhance the understanding of oral frailty.

Frailty is a state in old age characterized by heightened susceptibility to stress because of diminished physiological reserve.^3^ It predisposes individuals to various adverse outcomes, including functional disability, long‐term nursing care, elevated healthcare costs, and increased mortality.^4^ Frailty can be reversed through early identification and implementation of appropriate interventions.^3^ Frailty is also multifaceted, encompassing physical issues (muscle strength loss, leading to increased fall risks), mental and psychological issues (cognitive impairment and depression), and social issues (living alone, social ties, and economic deprivation). Recently, recognition of its multifaceted aspects has increased, including oral function. These aspects can interact to form a negative chain of events resulting in functional disability and death.^3^

Effective frailty prevention at an early stage is challenging. Countermeasures must go beyond healthcare professionals' assessments and interventions, requiring early awareness, personal engagement, and multidisciplinary coordination.^3^ The 2016 “National Council for Promoting Active Engagement of All Citizens” includes measures to address frailty, emphasizing reinforcing efforts in nutrition, oral health, exercise, and medications. The importance of frailty countermeasures has been highlighted in the “Integrated Implementation of Health Service and Preventive Long‐Term Care Service for the Elderly” initiated by the Ministry of Health, Labor and Welfare in Japan (MHLW) in 2020.^5^ Within this initiative, a newly introduced 15‐question frailty checkup includes two questions on oral function.^5^

## Origins and evolution of the concept of oral frailty

The concept of oral frailty was first proposed in 2014 in Japan.^6^ Subsequent research has been conducted, leading to the formulation of the present statement. The “8020 Campaign,” initiated in Japan in 1989, is the most popular oral health initiative with the highest public participation among older adults; it aims for individuals to maintain at least 20 natural teeth by the age of 80 years. By 2016, over 50% of the population had achieved this goal. The evolving oral health needs of older adults have necessitated substantial changes in oral health initiatives. The MHLW in Japan put forward concepts such as the “Future Projections of Dental Care Demand” and “Vision of Dental Health Care,” culminating in a preliminary proposal in 2017.^7^ In 2006, measures were implemented to minimize the onset of conditions necessitating long‐term care, marking a shift from disease prevention to extending healthy life expectancy. As part of long‐term care prevention, the oral health improvement service, which incorporates oral function assessment, was introduced.^8^ In 2014, the “Dental Health Examination for Latter‐Stage Older Adults” received government subsidies^9^ to screen focused on oral function, introducing the oral frailty checkup. In 2018, “oral hypofunction” became a new medical condition covered by the National Health Insurance, incorporating a comprehensive oral function examination, including measurements of bite force, tongue pressure, and masticatory function.^10^

There has been a significant shift in oral health initiatives, leading to the implementation of public frameworks in long‐term care insurance and National Health Insurance that emphasize oral function. Based on time‐series insurance data (including outcomes such as sarcopenia, frailty, long‐term care need certification, and mortality), Tanaka *et al*. presented impactful findings that oral frailty was statistically significantly associated with healthy life expectancy,^11^ sparking an intensified discourse on oral frailty. The Japan Dental Association deliberated nationwide measures against oral frailty in 2018, creating a leaflet for the general public.^12^ In 2019, the “Manual on Implementing Oral Frailty Countermeasures in Dental Clinics (2019)” was created,^13^ providing a clear definition for oral frailty. In 2020, the “Manual on Implementing Oral Frailty Countermeasures in Community Settings: Towards Integrated Implementation of Healthcare Services and Long‐term Care Need Prevention for Older Adults (2020)” was created,^14^ expanding oral frailty countermeasures at the municipal and health center levels, and a specific training program was presented.^15^

## Joint working group on oral frailty

In 2022, the “Joint Working Group on Oral Frailty” was established by the Japan Geriatrics Society, the Japanese Society of Gerodontology, and the Japanese Association on Sarcopenia and Frailty, aiming to further public understanding of oral frailty. The trends mentioned are addressed in the conference report by the Japanese Association for Dental Science defining “oral health management” and “oral frailty.”^16^ According to the report, the distinction between “age‐related decline in oral function” and “oral frailty” lies in the presence or absence of reversible factors within the progressive process. The senile process is a progressive natural process that cannot be avoided. Oral frailty is a multifactorial decline in oral function caused by multiple factors, including physical, social, mental, and cognitive domains. This is the difference between aging and oral frailty. Oral frailty is a multifactorial state with various treatment methods. Early identification of oral frailty and implementation of appropriate interventions can mitigate and possibly reverse oral frailty. The Oral Frailty 5‐item Checklist (OF‐5) presented here is anticipated to serve as an important instrument in facilitating these interventions.

## Aim of this statement

Oral frailty, marked by slight declines in various oral functions, including tooth loss, may increase the risk of impairment of oral function. With proper intervention and treatment, it could be improved.^17^, ^18^ It may lead to physical frailty, muscle weakening (sarcopenia), and undernutrition. We aim to contribute to the prevention and mitigation of oral frailty through enhancing multidisciplinary collaboration involving medical and dental healthcare professionals. The concurrent presence of oral function decline with frailty and other comorbidities elevates the increased risk of functional disability and death.^11^, ^19^ Thus, we propose with this consensus statement the following new concept: the definition of “oral frailty.”

**Concept of oral frailty**

Oral frailty is a state of oral function between the normal state of a “healthy mouth” and the “decline of oral function.”

**Definition of oral frailty**

Oral frailty is characterized by the accumulation of slight declines in oral function, including tooth loss and difficulties in eating and communicating, which increases the risk of impaired oral functional capacity. However, proper intervention and treatment can effectively improve this condition.

**Intention of this consensus statement**

**Figure 1:**
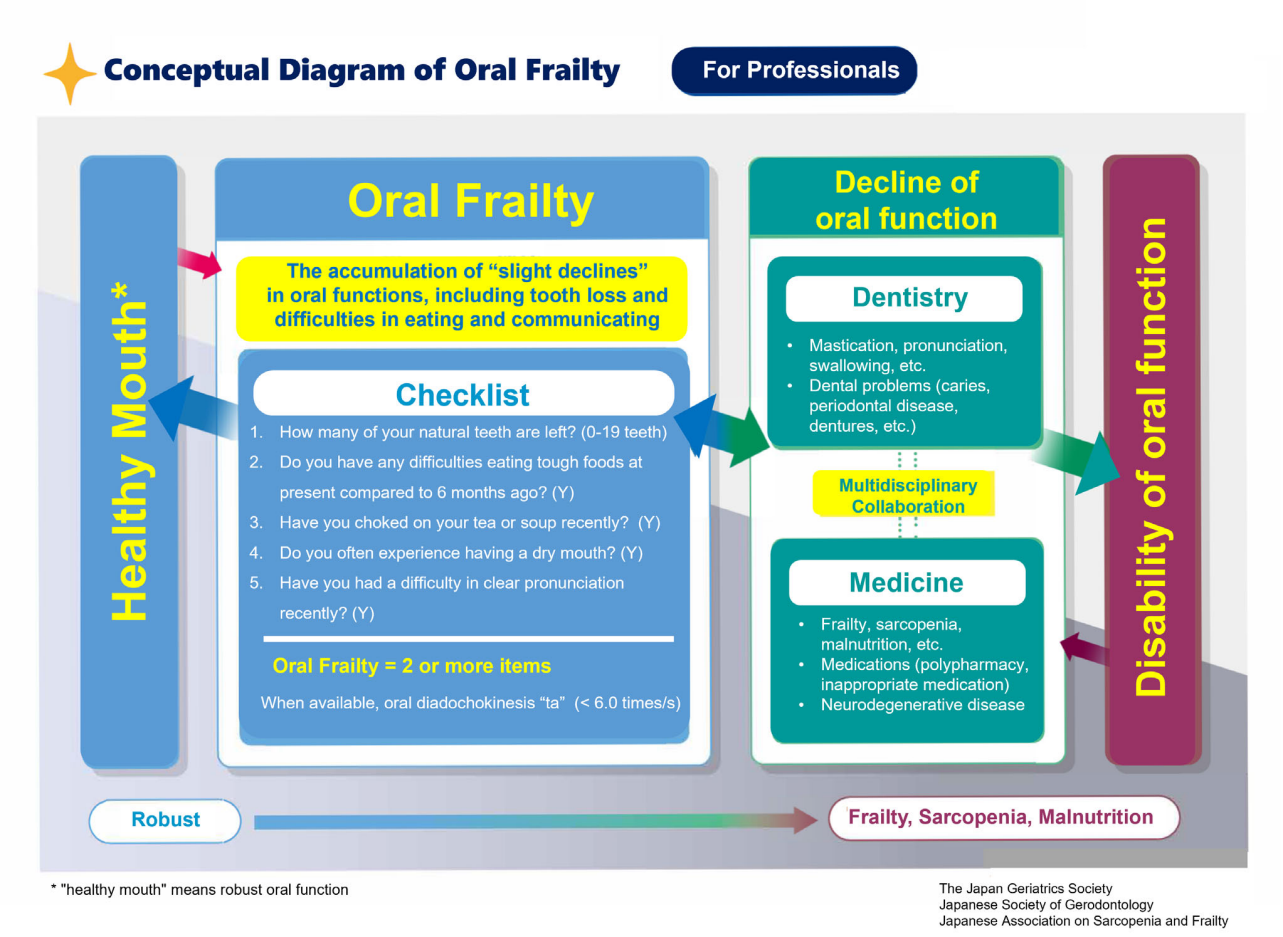
Conceptual diagram of oral frailty for healthcare professionals.

**Figure 2:**
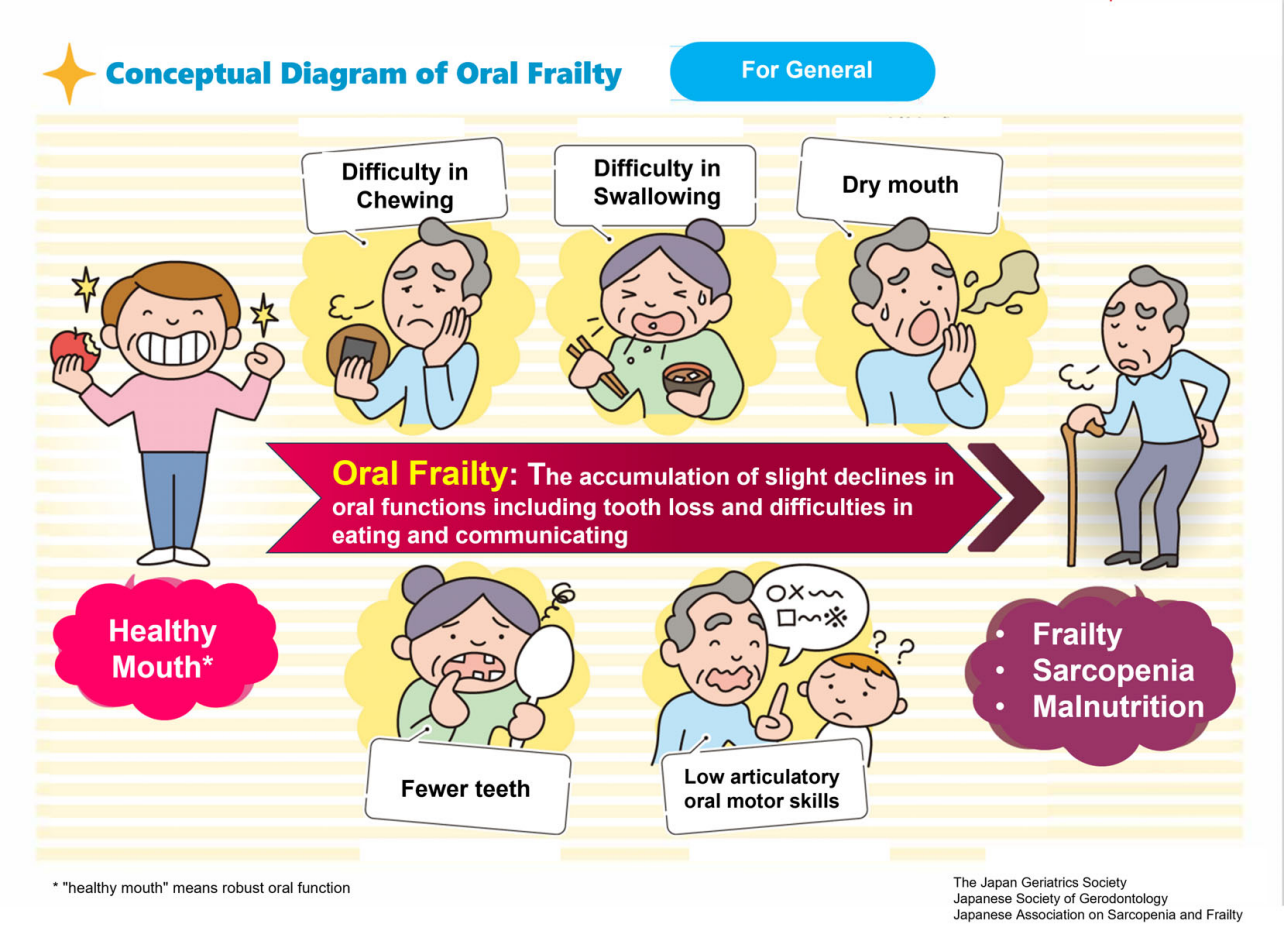
Conceptual diagram of oral frailty for the general public.

This consensus statement is intended to facilitate a better understanding of slight declines in various oral functions from an early stage and promote public awareness, using the new concept and definition of oral frailty based on the multifaceted phenotype model of frailty and two conceptual diagrams (Figs 1 and 2).

## Conceptual diagrams of oral frailty for healthcare professionals and the general public

**Figure 3:**
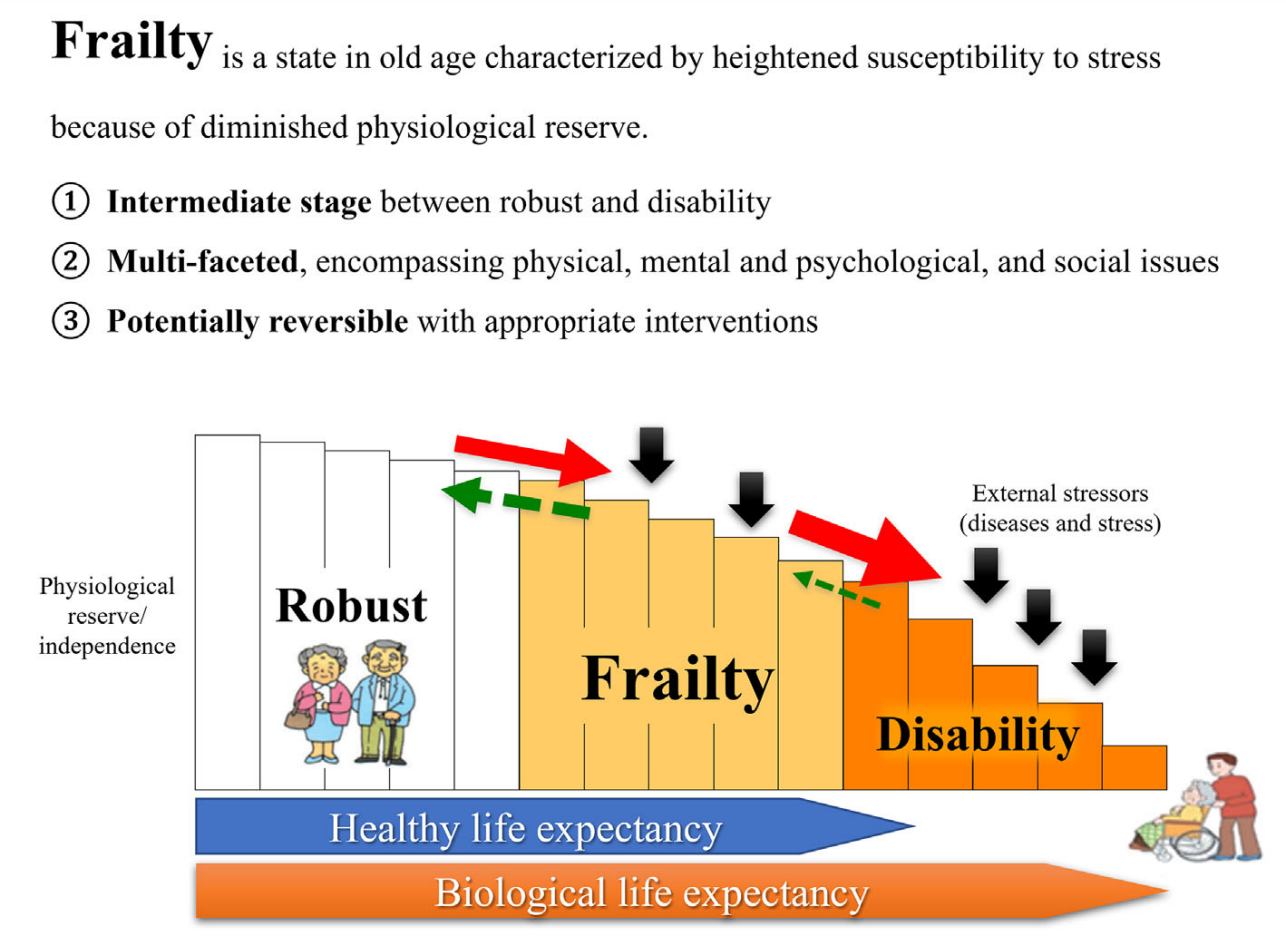
The Japanese concept of frailty. Modified from the literature “Masafumi Kuzuya. Impact of sarcopenia and frailty on elderly health. *Jpn J Geriatr*. 46(4), 279–285, 2009”.

Early alerts of slight declines in oral function, including tooth loss, require increased awareness and prevention. It is important for professionals in healthcare, nursing, and social welfare, including dental and medical practitioners, to share a common understanding and actively foster a transformation in awareness and behavior. For this purpose, we developed two distinct conceptual diagrams: one tailored for healthcare practitioners and another designed for the general public (Figs 1 and 2). These depict the progression from a healthy state of oral function to oral frailty and further to frailty, sarcopenia, and undernutrition (from left to right; Fig. 1). This diagram is based on the conceptual diagram of frailty in Japan (Fig. 3). The diagram for the general public presents these criteria in an accessible manner, while that for healthcare practitioners features the OF‐5 for practical evaluation. Addressing issues related to oral function and dental health requires the direct involvement of dental healthcare professionals, such as dentists or dental hygienists, and multidisciplinary collaborative interventions to address systemic health conditions, such as frailty, sarcopenia, and malnutrition, as well as their medication‐related risk factors, such as polypharmacy and potentially inappropriate prescriptions. Strategies for addressing neurodegenerative diseases are also crucial. Led by dental healthcare professionals such as dentists or dental hygienists, multidisciplinary collaboration can address decline of oral function to effectively halt the progression to disability of oral function, frailty, sarcopenia, and undernutrition. The diagrams and new checklist (OF‐5) proposed here enable early‐stage intervention against oral frailty in various settings.

## Assessment of oral frailty

Based on the evidence presented below (Evidences #1 to #3),^19^, ^20^, ^21^ we developed the OF‐5 for assessing oral frailty that can be performed even without dental healthcare professionals such as dentists or dental hygienists (Table 1). Oral frailty is defined as at least having two of the following five components: (i) fewer teeth, (ii) difficulty in chewing, (iii) difficulty in swallowing, (iv) dry mouth, and (v) low articulatory oral motor skills (question about self‐perceived difficulty with clear pronunciation). Questions regarding three oral functions (difficulty in chewing, difficulty in swallowing, and dry mouth) were formulated by selecting questions from the Kihon Checklist, which is used extensively in Japan to identify older individuals at risk of requiring long‐term support or care. Joint Working Committee on Oral Frailty by the Japan Geriatrics Society, Japanese Society of Gerodontology, and Japanese Association on Sarcopenia and Frailty expect/‘anticipate’ translations of the Oral Frailty 5‐item Checklist (OF‐5). In addition, we would like to have one official translated version of the OF‐5 per language, produced through a scientific process. If you are interested in the official translation of the OF‐5 into your language, please contact the working group. In the list below, languages marked with “In preparation for publication” are already in the process of being translated (https://www.gerodontology.jp/committee/002376.shtml). Currently, evidence on oral frailty evaluated using the OF‐5 is primarily derived from Japanese studies. As more evidence accumulates in the future, it will be important to account for international regional differences, including cultural attitudes, to apply the criteria outlined in this statement globally.

**Table 1:** Oral frailty five‐item checklist

Component	Questionnaire item	Response	
		Applicable	Not applicable
Fewer teeth	How many of your natural teeth are left?	0–19 teeth	≥20 teeth
Difficulty in chewing	Do you have any difficulties eating tough foods compared with 6 months ago?	Yes	No
Difficulty in swallowing	Have you choked on your tea or soup recently?	Yes	No
Dry mouth	Do you often experience having a dry mouth?	Yes	No
Low articulatory oral motor skill^†^	Have you had difficulty with clear pronunciation recently?	Yes	No

Component	Measurement	Response	
		Applicable	Not applicable
Low articulatory oral motor skills^†^	ODK with/ta/	<6.0 times/s	≥6.0 times/s

Oral frailty is characterized by the presence of two or more of the above five components.

^†^ Measurement of the repetitive articulatory rate or ODK is a reliable method of articulatory oral motor skill. In addition to the five components above of the oral frailty five‐item checklist, ODK can be measured outside healthcare settings using specific devices or applications.

ODK, oral diadochokinesis.

Based on the OF‐5, approximately 40% of community‐dwelling older adults met the criteria for oral frailty (Evidences #1 and #3).^19^, ^21^ Older adults with oral frailty based on the OF‐5 had poor dietary variety and social isolation, physical frailty, long‐term care certification need, and increased mortality (Evidences #1 and #3).^19^, ^21^ A novel questionnaire‐based assessment for articulatory oral motor skills within the OF‐5 has been validated (Evidence #2).^20^ A detailed explanation procedure for the English translation of the OF‐5 is provided in the supporting information for Evidence #3.^20^ This tool, requiring no specialized equipment or techniques, allows versatile community usage, raising awareness about the deterioration of oral health at earlier stages. Its application beyond dentistry in medical institutions would enhance oral frailty countermeasures through multidisciplinary collaboration and is anticipated to facilitate diverse initiatives tailored to improving overall health status and preventing declining eating ability in community‐dwelling older adults, helping reverse and prevent oral frailty and promoting stable nutritional management. In the future, systematic and feasibility research on specific interventions to prevent or improve oral frailty will be necessary, with an emphasis on the practicality of these interventions in clinical practice and community settings. The following is a summary of evidence developed by this working committee:

## Evidence #1: Longitudinal association of Oral Frailty 5‐item Checklist with adverse health outcomes

### Objective

This study aimed to elucidate the prevalence of oral frailty using the OF‐5 and its association with frailty, the need for long‐term care, and mortality.

### Study participants and methods

Longitudinal data were collected from a prospective cohort study involving 2044 randomly selected community‐dwelling independent older adults in a specific municipality. Following a baseline survey conducted in 2012, subsequent follow‐up surveys were carried out in 2013, 2014, 2016, 2018, and 2021. Frailty was assessed using the phenotypic model proposed by Fried *et al*.,^4^ and public information from government sources was used to obtain care‐related data, including the presence and timing of care requirements. The OF‐5 was administered using a designated questionnaire and an oral diadochokinesis (ODK) test on the articulation of the “ta” syllable.

### Results

Among the 2031 participants analyzed (mean age 73.1 ± 5.6 years; 51.1% women), the prevalence of oral frailty was 39.3%. Oral frailty was defined as meeting at least two criteria in the OF‐5, based on the significant increase in both the prevalence and incidence rates of frailty observed with these criteria.

Older adults with oral frailty exhibited a considerably higher prevalence of frailty at the time of the study, as well as markedly elevated hazard rates for the new onset of frailty, long‐term care need, and mortality during follow‐up for up to 9 years. These associations remained notable even after adjusting for factors such as age and pre‐existing conditions.

**Figure 4:**
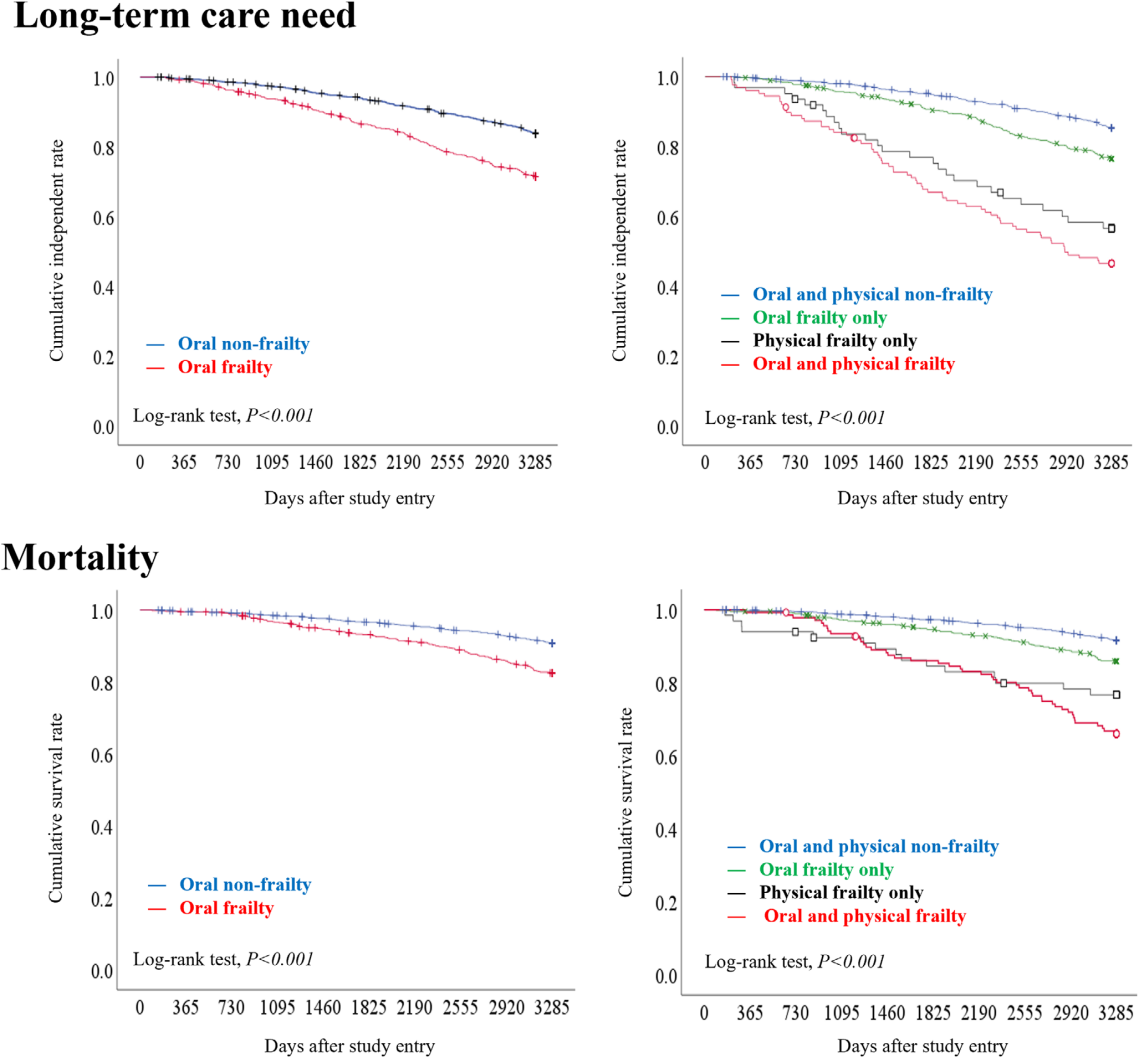
Kaplan–Meier curves for long‐term care needs and mortality stratified by oral frailty status and oral and physical frailty status at baseline.^19^

Upon stratifying by the frailty status in the baseline survey and examining its association with the need for long‐term care and mortality, we found that the hazard ratio was markedly elevated in frail older adults with concurrent oral frailty (Fig. 4). Even in non‐frail older adults, oral frailty was associated with a considerably higher hazard ratio.

### Findings

Oral frailty, assessed by the OF‐5, serves as a predictor for frailty, long‐term care needs, and mortality among community‐dwelling older adults. The OF‐5 is a novel assessment tool that can be administered irrespective of setting. It contributes to oral frailty countermeasures through multidisciplinary collaboration, primarily involving medical and dental healthcare professionals.

## Evidence #2: Validation of self‐reported articulatory oral motor skills

### Objective

ODK, which involves the rapid repetition of monosyllables (such as “pa,” “ta,” and “ka”) and the measurement of the number of utterances per second, serves as an objective method for assessing articulatory oral motor skills. Despite being well‐established, implementation can be challenging, particularly without the required equipment. In this study, new questions on articulatory oral motor skills were developed and validated to address potential implementation issues.

### Study participants and methods

In 2022, a questionnaire on articulatory oral motor skills was administered among the participants of a comprehensive health examination conducted by a Japanese research institution. The survey posed the question, “Have you had difficulty with clear pronunciation recently?” (Yes/No). This survey was carried out twice, approximately 4 weeks apart. Furthermore, an ODK testing the articulation of the “ta” syllable was conducted. Using the two sets of responses obtained from the questionnaire, kappa coefficients were computed to assess the test–retest reliability. Participants who responded “Yes” were classified as having self‐perceived low articulatory oral motor skills, and a comparison was made between the articulation of the “ta” syllable in the ODK test and other characteristics based on the presence or absence of the self‐perceived low articulatory oral motor skills.

### Results

Six hundred and seven participants (mean age, 73.9) were included in the analysis, and 18.5% were classified as having a self‐perceived decline in articulatory oral motor skills. The derived kappa coefficient was 0.71, a significantly high value.

Participants with self‐perceived low articulatory oral motor skills had a markedly lower repetitive articulatory rate for the “ta” syllable compared with those without this perception. They also reported a higher prevalence of functional decline in chewing and swallowing, along with increased oral dryness. A large proportion of these participants were classified as physically frail (Table 2). The questions exhibited high specificity (83.1%) in detecting an objective decline in articulatory oral motor skills based on the ODK test, although the sensitivity was low (42.1%) (Table 3).

**Table 2:** Characteristics of study participants according to self‐reported articulatory oral motor skill

	Total (*N* = 607)	Have you had difficulty with clear pronunciation recently?		*P‐*value
		No (*N* = 495)	Yes (self‐reported low articulatory oral motor skill) (*N* = 112)	
ODK with/ta/ (times/s)	6.5 ± 0.8	6.5 ± 0.7	6.3 ± 1.0	<0.01
Difficulty in chewing	155 (25.5)	104 (21.0)	51 (45.5)	<0.01
Difficulty in swallowing	162 (26.7)	120 (24.2)	42 (37.5)	<0.01
Complaint of dry mouth	153 (25.2)	113 (22.8)	40 (35.7)	0.01
Physical frailty	39 (6.4)	27 (5.5)	12 (10.7)	0.04

Data are presented as mean ± SD or *n* (%).

ODK, oral diadochokinesis.

**Table 3:** Verification of the validity of questions regarding articulatory oral motor skills

	Total (*N* = 607)	Have you had difficulty with clear pronunciation recently?		*P‐*value	Sensitivity (95% CI)	Specificity (95% CI)
		No (*N* = 495)	Yes (self‐reported low articulatory oral motor skill) (*N* = 112)			
Low articulatory oral motor skill based on ODK performance^†^	38 (6.3%)	22 (4.4%)	16 (14.3%)	<0.01	42.1% (26.3%–59.2%)	83.1% (79.8%–86.1%)

^†^ Presented as *n* (%).

ODK, oral diadochokinesis; CI, confidence interval.

### Findings

The newly developed questions on articulatory oral motor skills demonstrated adequate test–retest reliability and a remarkable association with the ODK test results, which is an objective method of assessing articulatory oral motor skills.

## Evidence #3: Prevalence of oral frailty determined by the Oral Frailty 5‐item Checklist

### Objective

To assess the prevalence of oral frailty using the OF‐5 and examine its associations with dietary variety, social engagement, and physical frailty among community‐dwelling older adults.

### Study participants and methods

**Figure 5:**
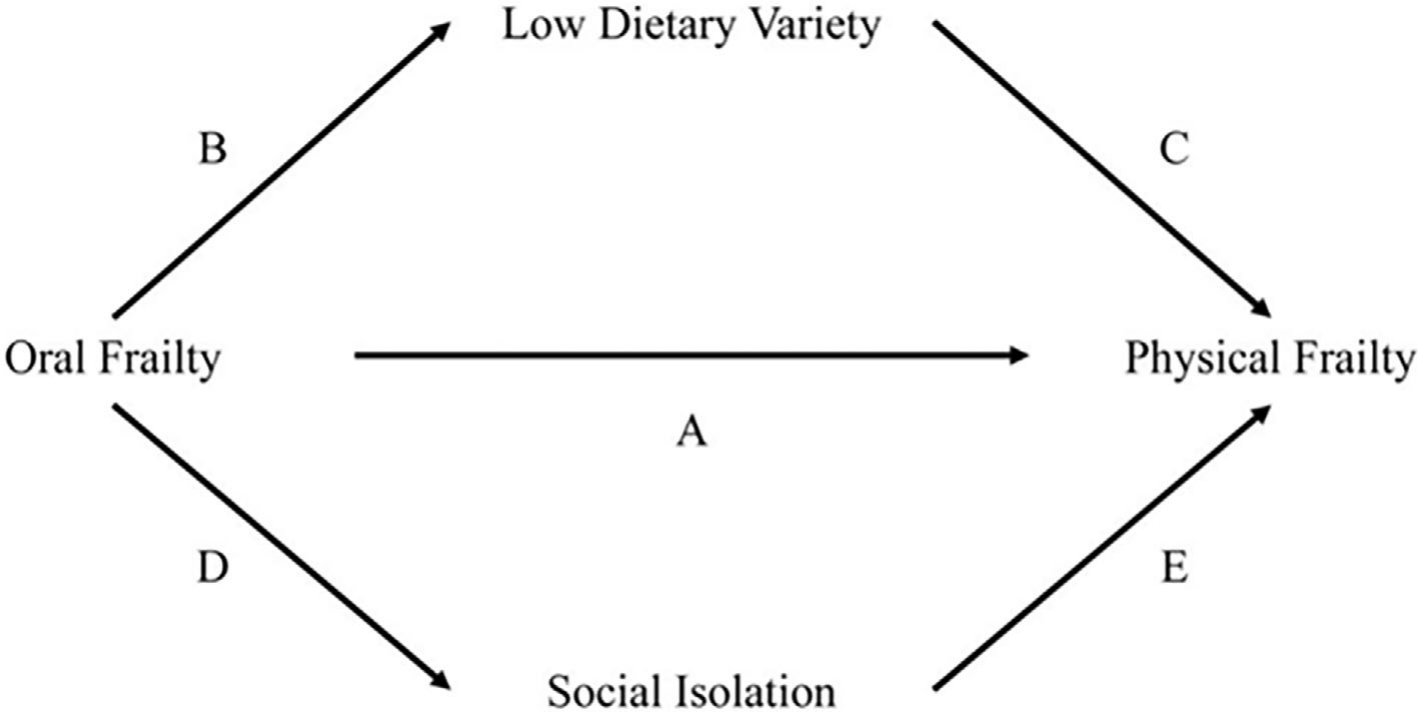
Associations between oral frailty, low dietary diversity, social isolation, and physical frailty.^21^

Participants of a comprehensive health examination conducted by a domestic research institute in 2022 were included. Oral frailty was assessed using the OF‐5 and was defined as having two or more of the following five components: (i) fewer teeth, (ii) difficulty in chewing, (iii) difficulty in swallowing, (iv) dry mouth, and (v) low articulatory oral motor skills. Dietary variety was evaluated using the dietary variety score, with a score of ≤3 defined as low variety. Social interaction was assessed using the Lubben Social Network Scale‐6, with a score <12 defined as social isolation. Physical frailty was assessed using the revised Japanese Cardiovascular Health Study criteria, with ≥3 criteria defined as physical frailty. Whether oral frailty affects physical frailty through low dietary diversity and social isolation was determined using structural equation modeling to estimate direct (pathway A in Fig. 5) and indirect effects (pathways BC and DE in Fig. 5).

### Results

Of the 1206 participants in the analysis (mean age 74.7 years), 36.7% had oral frailty. The rate of oral frailty increased with age, but there was no difference between men and women. Oral frailty indirectly affected physical frailty through low dietary diversity and social isolation (pathways BC and DE in Fig. 5) (odds ratio for physical frailty due to low dietary diversity = 1.43, 95% confidence interval = 1.04−1.97; odds ratio for physical frailty due to social isolation = 1.42, 95% confidence interval = 1.04−1.94). The direct effect of oral frailty on physical frailty (pathway A in Fig. 5) was not statistically significant (odds ratio = 1.14, 95% confidence interval = 0.65−1.98).

### Findings

Approximately 40% of community‐dwelling older adults had oral frailty as assessed using the OF‐5. Oral frailty was indirectly associated with physical frailty via low dietary variety and social isolation.

## Conclusion

This statement introduces the concept and definition of oral frailty. Along with a newly developed assessment tool and two concept maps for healthcare professionals and the general public, we propose that the concept and definition of oral frailty be used to further increase public awareness and promote multidisciplinary collaboration.

## Disclosure statement

The authors declare no conflicts of interest.

## Supporting information


**Data S1.** Supporting Information.

## Data Availability

Data sharing not applicable to this article as no datasets were generated or analysed during the current study.
