# Supplementary material for: Consensus statement on “Oral frailty” from the Japan Geriatrics Society, the Japanese Society of Gerodontology, and the Japanese Association on Sarcopenia and Frailty
Source: Geriatr Gerontol Int. 2024 Oct 7;24(11):1111–9. doi: 10.1111/ggi.14980 (PMC11843523; doi:10.1111/ggi.14980)
Supplement: Supplementary file 1 — Data S1. Supporting Information. [file GGI-24-1111-s001.docx]

**Supporting Information**

**English translation of the Oral Frailty 5-item Checklist (OF-5)**

Three of five components (i.e., questionnaire items) of the OF-5, namely, (ii) difficulty in chewing, (iii) difficulty in swallowing, and (iv) dry mouth, are identical to those in the “Kihon Checklist” (KCL). English translation of the KCL has been performed by Arai and Satake^1^ in a reliable manner; therefore, our English translation work will cover the remaining two components: (i) fewer teeth and (v) low articulatory oral motor skills. The translation process of the original Japanese version (JV1) to the English version consisted of the following five stages:

**Stage 1.** The translation of the JV1 into English was performed by three independent people who were involved in the "Joint Working Committee on Oral Frailty" by three academic societies—the Japan Geriatrics Society, the Japanese Society of Gerodontology, and the Japanese Association on Sarcopenia and Frailty—who were proficient in English and who were specialists of the Japanese Society of Gerodontology or Japanese Society for Oral Health. Consequently, three independent test versions (T1, T2, and T3) were created.

**Stage 2.** A preliminary version (the first English version of the OF-5 components; EV1) was synthesized by combining the best cultural and clinical translation of each item by T1, T2, and T3 together. This process was performed by an expert committee (composed of M.I., T.T., K.I., T.U., K.I., and H.H.).

**Stage 3.** One independent native-speaking Japanese specialist in gerodontology back-translated the EV1 from English to Japanese, and the back-translated version (JV2) was created.

**Stage 4.** An expert committee ensured that the JV2 reflected the same item content (literal, conceptual, and semantic equivalence) as the JV1. At this stage, several components had to go through the earlier stages repeatedly until an expert committee provided a final certification. This process was used to revise the EV1 and construct the English version 2 (EV2).

**Stage 5**. One independent native English-speaking certified translator confirmed the expression of EV2. Then, we approved the final version of the OF-5. This version is presented in Table S1.

**Assessment of dietary variety**

The dietary variety of participants was assessed using the dietary variety score (DVS).^2^ The DVS is determined by assessing the number of food groups (meat, fish/shellfish, eggs, soy products, milk, green/yellow vegetables, seaweed, potatoes, fruit, and fats/oils) that are consumed at least once a day. The DVS ranges from 0 to 10, with lower scores indicating lower dietary variety.^2^ According to previous studies,^3,4^ low dietary variety was defined as a DVS ≤3.

**Assessment of social engagement**

Participants’ social engagement was assessed using the Japanese version of the Lubben Social Network Scale (LSNS-6).^5,6^ The LSNS-6 contains three questions that evaluate social connectedness with relatives and three questions that evaluate social connectedness with friends. The LSNS-6 score has a range of 0–30, with lower scores indicating greater social isolation. As suggested by Lubben et al.,^6^ we defined social isolation as an LSNS-6 score <12.

**Assessment of physical frailty**

Based on the information obtained through questionnaires and physical evaluations, physical frailty was assessed using the Japanese version of the Cardiovascular Health Study (J-CHS) criteria.^7^ The J-CHS criteria included the following five components. (i) “Shrinking” was indicated by a response of “yes” to the question, “Have you lost 2 kg or more in the past 6 months?”. (ii) “Weakness” was indicated by grip strength <28 kg in men or <18 kg in women. (iii) “Exhaustion” was indicated by a response of “yes” to the question, “In the past 2 weeks, have you felt tired without a reason?”. (iv) “Slowness” was indicated by a normal gait speed <1.0 m/s. (v) “Low activity level” was indicated by responses of “no” to both of the following questions: “Do you engage in moderate levels of physical exercise or sports aimed at health?” and “Do you engage in low levels of physical exercise aimed at health?” Each component is allocated one point if present. Overall, the J-CHS score ranges from 0 to 5. Participants with a J-CHS score ≥3 were defined as exhibiting physical frailty.^7^

**REFERENCES**

1. Arai H, Satake S. English translation of the Kihon Checklist. Geriatr Gerontol Int. 2015 Apr;15(4):518–9.

2. Kumagai S, Watanabe S, Shibata H, Amano H, Fujiwara Y, Shinkai S et al. Effects of dietary variety on declines in high-level functional capacity in elderly people living in a community. Nihon Koshu Eisei Zasshi. 2003 Dec;50(12):1117–24.

3. Yokoyama Y, Nishi M, Murayama H, Amano H, Taniguchi Y, Nofuji Y et al. Dietary variety and decline in lean mass and physical performance in community-dwelling older Japanese: a 4-year follow-up study. J Nutr Health Aging. 2017 Apr 21;21(1):11–6.

4. Narita M, Kitamura A, Takemi Y, Yokoyama Y, Morita A, Shinkai S. Food diversity and its relationship with nutrient intakes and meal days involving staple foods, main dishes, and side dishes in community-dwelling elderly adults. Nihon Koshu Eisei Zasshi. 2020;67(3):171–82.

5. Kurimoto A, Awata S, Ohkubo T, Tsubota-Utsugi M, Asayama K, Takahashi K et al. Reliability and validity of the Japanese version of the abbreviated Lubben Social Network Scale. Nihon Ronen Igakkai Zasshi. 2011 Jul;48(2):149–57.

6. Lubben J, Blozik E, Gillmann G, Iliffe S, Kruse WVR, Beck JC et al. Performance of an abbreviated version of the Lubben Social Network Scale among three European community-dwelling older adult populations. Gerontologist. 2006 Aug;46(4):503–13.

7. Satake S, Arai H. The revised Japanese version of the Cardiovascular Health Study criteria (revised J-CHS criteria). Geriatr Gerontol Int. 2020 Oct;20(10):992–3.

**Table S1.** Oral Frailty 5-item Checklist (OF-5; questions and responses shown in English)

| **Component** | **Questionnaire item** | **Response** | |
| --- | --- | --- | --- |
|  |  | **Applicable** | **Not applicable** |
| Fewer teeth | How many of your natural teeth are left? | 0–19 teeth | ≥20 teeth |
| Difficulty in chewing | Do you have any difficulties eating tough foods compared to 6 months ago? | Yes | No |
| Difficulty in swallowing | Have you choked on your tea or soup recently? | Yes | No |
| Dry mouth | Do you often experience having a dry mouth? | Yes | No |
| Low articulatory oral motor skill^*^ | Have you had a difficulty in clear pronunciation recently? | Yes | No |

Oral frailty is characterized by the presence of two or more of the above five components.

^*^The measurement of the repetitive articulatory rate or oral diadochokinesis (oral DDK) is a reliable measure for articulatory oral motor skill. Using specific devices or applications, oral DDK can be measured outside healthcare settings. In addition to the above 5 components of OF-5, oral DDK measurements are recommended to be performed.

| **Component** | **Measurement** | **Response** | |
| --- | --- | --- | --- |
|  |  | **Applicable** | **Not applicable** |
| Low articulatory oral motor skill* | Oral DDK with/ta/ | <6.0 times/s | ≥6.0 times/s |

**Table S2.** Oral Frailty 5-item Checklist (OF-5; questions and responses shown in Japanese)

| **項目** | **質問** | **選択肢** | |
| --- | --- | --- | --- |
|  |  | **該当** | **非該当** |
| 歯数減少 | 自身の歯は、何本ありますか  （さし歯や金属をかぶせた歯は、自分の歯として数えます。インプラントは、自分の歯として数えません。） | 0～19本 | 20本以上 |
| 咀嚼困難感 | 半年前と比べて固いものが食べにくくなりましたか | はい | いいえ |
| 嚥下困難感 | お茶や汁物等でむせることがありますか | はい | いいえ |
| 口腔乾燥感 | 口の渇きが気になりますか | はい | いいえ |
| 滑舌低下（口唇・舌の巧緻性の低下）^*^ | 普段の会話で、言葉をはっきりと発音できないことがありますか | はい | いいえ |

**5つの項目のうち、2つ以上に該当する場合をオーラルフレイル**

^*^滑舌低下について：舌口唇運動機能（巧緻性および速度）の検査であるオーラルディアドコキネシスは、医療機関ではない場所でも、簡便な測定装置もしくはアプリケーションで実測が可能であり、上記5項目に加えて測定されることが望まれる。

| **項目** | **計測** | **滑舌低下** | |
| --- | --- | --- | --- |
|  |  | **該当** | **非該当** |
| 滑舌低下（口唇・舌の巧緻性の低下）* | オーラルディアドコキネシス（タ音の1秒当たりの発音回数） | 6.0回/秒未満 | 6.0回/秒以上 |
